# Investigation of glacier mass balance based on multi-source observation in the Karakoram over the past 25 years

**DOI:** 10.1371/journal.pone.0358980

**Published:** 2026-10-09

**Authors:** Aijie Yu, Hongling Shi, Hubiao Wang, Zhiping Jiao, Ping Wang, Chunchun Gao, Harald Schuh

**Affiliations:** 1 Innovation Academy for Precision Measurement Science and Technology, Chinese Academy of Sciences, Wuhan, China; 2 University of Chinese Academy of Sciences, Beijing, China; 3 Institute of Geodesy and Geoinformation Science, Technische Universität Berlin, Berlin, Germany; 4 Henan Remote Sensing Institute, Zhengzhou, China; 5 College of Geodesy and Geomatics, Shandong University of Science and Technology, Qingdao, China; 6 Geodesy, GFZ Helmholtz Centre for Geosciences, Potsdam, Germany; Swedish Meteorological and Hydrological Institute, SWEDEN

## Abstract

The “Karakoram Anomaly” — the atypical stability of glaciers in a warming climate—has introduced considerable uncertainty into projections of regional water resources. However, the resilience of this anomaly under sustained climate forcing remains poorly quantified. In this study, multi-source geodetic data (ASTER, NASA DEM, ICESat‑2) were integrated to reassess glacier mass balance across the Karakoram and its sub‑regions between 2000 and 2024. Our results indicate a region‑wide specific mass balance of −0.0386 ± 0.0032 m w.e. a ^−^ ¹, with strong spatio-temporal heterogeneity. We document a progressive decay of the Karakoram Anomaly: it was region‑wide before 2010, contracted to the northern and central Karakoram during 2010–2020, and has largely weakened since 2020, persisting only in isolated basins. This evolution is closely associated with summer warming and changes in precipitation. Topography exerts a key control on spatial variability. These findings improve our understanding of the Karakoram anomaly and are of great significance for regional water resources management and disaster prevention under climate change.

## 1. Introduction

Glacier mass balance is a fundamental and sensitive indicator of climate change, with profound implications for global sea-level rise, regional water security, and geohazard dynamics [1]. Recent global assessments consistently underscore the scale of contemporary ice loss: glaciers worldwide lost mass at a rate of 267 ± 16 Gt/a between 2000 and 2019, contributing 21 ± 3% to observed sea-level rise [2]. This accelerated wastage triggers cascading consequences, including freshwater scarcity and an increasing occurrence of abrupt hazardous events such as ice avalanches, debris flows, and glacier lake outburst floods (GLOFs). A robust assessment of glacier mass balance is therefore essential for understanding glacier response to climate change and anticipating future water-resource and glacier-related hazards.

The High Mountain Asia (HMA) region contains the largest concentration of glaciers outside the polar regions and provides critical water resources for several major Asian river systems, including the Yangtze and Yellow Rivers in China and numerous transboundary rivers in South and Southeast Asia. However, HMA is experiencing rapid cryospheric change under accelerated warming, with surface temperatures increasing at a rate of 0.3 ± 0.2 °C per decade, exceeding the global mean warming rate of 0.2 ± 0.1 °C per decade [3]. This pronounced warming has resulted in widespread glacier retreat and enhanced mass loss across the region, with profound implications for global sea-level rise, regional water availability, ecosystem stability, and cryosphere-related hazards [4]. Therefore, accurately quantifying glacier mass balance in HMA is essential for understanding glacier–climate interactions and assessing future water-resource and hazard risks.

In contrast to the widespread glacier retreat observed across most of the Himalaya and Tibetan Plateau, glaciers in the Karakoram exhibit a distinct and heterogeneous response to climate change. This phenomenon, commonly referred to as the “Karakoram Anomaly” [5], is characterized by relatively stable or slightly positive mass balance, limited glacier shrinkage, enhanced ice-flow activity, and frequent surge-type glacier dynamics. Previous studies have attributed this anomaly to a combination of regional climatic and glaciological factors, including relatively cooler summer temperatures, increased winter snowfall, and dynamic glacier responses.

Despite extensive investigations, the persistence and evolution of the Karakoram Anomaly remain unresolved. Some studies suggest that the anomaly has persisted into the 2010s and even the 2020s, with glaciers maintaining near-equilibrium mass balance in recent decades [6], whereas others indicate that the anomaly has weakened or transitioned toward net mass loss, particularly after 2015 [2]. Moreover, the timing and spatial pattern of anomaly evolution remain debated, with some studies suggesting an earlier decline around 2010–2015, while others report continued positive mass balance in parts of the western Karakoram into the 2020s, implying possible regional differences in the decay of the anomaly [7]. The recent anomalous trends in the Karakoram and the heterogeneity of glacier mass balance across its various sub-regions remain unclear. Addressing these questions is particularly important because the Karakoram hosts numerous surge-type glaciers and rapidly evolving glacier lakes, which can alter downstream runoff regimes and increase the potential risk of glacier-related hazards such as glacier lake outburst floods (GLOFs). Therefore, an updated, high-resolution assessment of glacier mass balance across the entire Karakoram is urgently needed.

Current glacier mass change estimation relies on four complementary approaches: glaciological measurements (stake networks, snow probing, UAV surveys), geodetic DEM differencing, satellite altimetry (e.g., ICESat‑1/2, CryoSat‑2), and satellite gravimetry (GRACE/GRACE‑FO) [2,8,9]. These approaches have been widely applied to investigate glacier mass changes in the Karakoram. However, these approaches provide different observational perspectives and suffer from limitations in spatial coverage, temporal continuity, or measurement uncertainty when applied individually. Recent studies in High Mountain Asia have demonstrated that integrating complementary satellite observations with climate and topographic information provides an effective framework for investigating glacier evolution [10,11].

Despite these advances, existing studies have mainly focused on long-term regional averages or estimates derived from individual observation systems. Consequently, the coupled evolution of glacier mass balance, spatio-temporal heterogeneity remains insufficiently understood. In this study, we integrate ICESat-2 observations with DEMs derived from ASTER stereo imagery and the SRTM DEM within a unified reference framework to investigate glacier mass changes across the Karakoram from 2000 to 2024. Specifically, this study aims to (1) investigate phased and high-resolution analysis of glacier mass balance spanning 2000–2024 and characterize the temporal evolution of the Karakoram anomaly; (2) quantify the spatial heterogeneity of glacier mass changes across 17 basins; and (3) assess the relative contributions of topographic and climatic factors to the observed spatial variability in glacier mass balance Overall, this study reveals a more detailed spatio-temporal evolution of glacier mass balance in the Karakoram region, providing an important reference for understanding the Karakoram Anomaly and regional water resource management.

## 2. Study area

The Karakoram Range, situated in Central Asia northwest of the Tibetan Plateau (33°–37°N, 73°–79°E), constitutes an integral component of the Himalayan-Kunlun cryospheric system (Fig 1). Extending across western China, northern Pakistan, and India, the region is characterized by extensive glaciation, covering an area of approximately 22,510 km^2^ with an average altitude of ~5,300 m. Estimates derived from published ice thickness data indicate a total glacier volume ranging from 2.03 × 10^3^ km^3^ (± 0.52 × 10^3^) to 2.81 × 10^3^ km^3^ (± 1.08 × 10^3^) [12]. The Karakoram region contains a high density of mountain glaciers, with nearly 10,500 individual glacier bodies, making it one of the most extensively glacierized regions in Asia. Several major valley glaciers, including the Siachen, Baltoro, and Biafo glaciers, exceed 50 km in length, a characteristic that is rare in mid- to low-latitude mountain regions worldwide. These glaciers play a critical role in regional hydrology, with glacier meltwater contributing approximately 48% of the total runoff of the Indus River and 53% of that of the Tarim River [13]. According to HydroBASINS dataset [14], the entire region can be further divided into 17 sub-basins.

**Fig 1:**
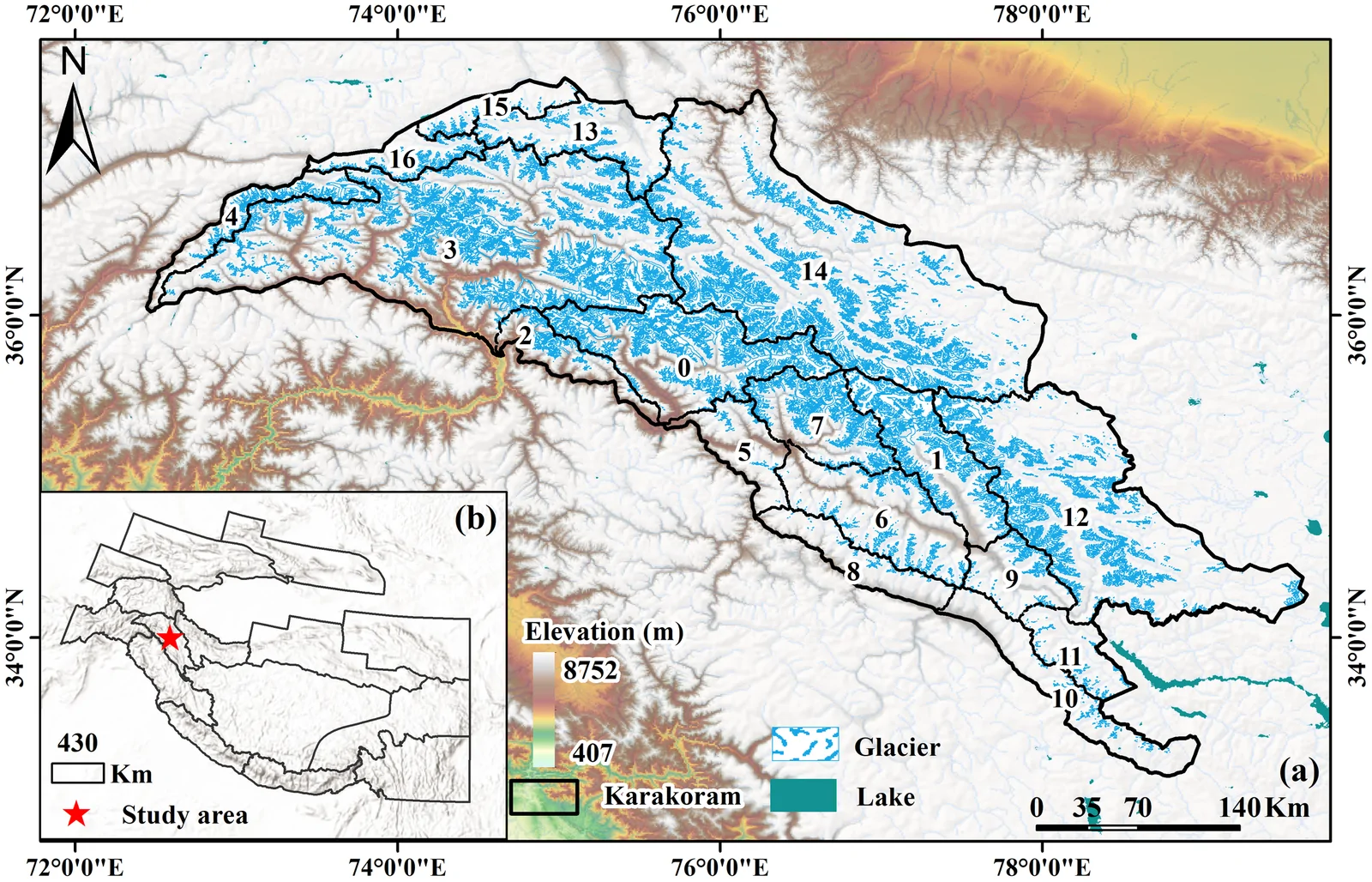
Location of the Karakoram region and the spatial distribution of glacier. (a) Distribution of different sub regions in the Karakoram; (b) the location of Karakoram in the HMA.Basemap data can be accessed via the https://services.arcgisonline.com/arcgis.

Climatically, the Karakoram is characterized by a semi-arid continental climate, with pronounced spatial heterogeneity in atmospheric circulation and moisture supply. The southern slopes are primarily influenced by the South Asian monsoon and receive substantially higher precipitation, whereas the northern slopes are dominated by westerly circulation and are comparatively drier. Air temperature exhibits a unimodal annual cycle, with minimum and maximum monthly means occurring in January and August, respectively. The region has a mean annual air temperature of about −24.8 °C and receives approximately 540 mm of precipitation per year [15].

## 3. Data and methods

### 3.1. Data

(1) Satellite altimetry data

Launched in September 2018, ICESat-2 continues the legacy of ICESat and is equipped with the ATLAS photon-counting lidar instrument, enabling high-resolution and high-accuracy surface elevation measurements that are particularly valuable for elevation change detection [16]. The satellite operates in a near-polar orbit at an altitude of ~496 km, with a 91-day repeat cycle. ATLAS employs a photon-counting lidar system consisting of three beam pairs (six beams in total), with each pair comprising a strong and a weak beam to optimize surface detection over varying reflectance conditions. The beam pairs are separated by ~3.3 km across track, while the strong and weak beams within each pair are spaced ~90 m across track and ~2.5 km along track. Along-track laser footprints are spaced at ~0.7 m, providing exceptionally dense sampling suitable for resolving fine-scale glacier surface elevation changes.

Compared with its predecessor, ICESat-2 represents a substantial advancement in spaceborne laser altimetry. It provides near-global coverage between 88° N and 88° S, substantially improved elevation accuracy, and markedly enhanced spatial resolution. The vertical precision of surface elevation retrievals reaches approximately 0.1 m, while the nominal footprint diameter is ~ 17.5 m [17]. These improvements enable ICESat-2 to resolve fine-scale glacier surface topography and elevation changes with high fidelity. Over a complete orbital cycle comprising 1,387 individual orbits, ICESat-2 achieves global surface coverage.

ICESat-2 observations have been widely applied in glacier studies, particularly across High Mountain Asia and the Tibetan Plateau, demonstrating their capability for resolving glacier surface elevation changes at regional scales [18,19]. In this study, we employed ICESat-2 Level-3A land ice elevation data (ATL06) spanning from the first quarter of 2019 to the third quarter of 2024 to derive time series of glacier surface elevation changes in the Karakoram region.

The ATL06 product provides high-density and high-precision surface elevation measurements, together with per-point quality flags and corrections for surface slope, geoid–ellipsoid reference, and relevant geophysical effects [20,21]. Following standard quality-control procedures, observations with non-zero quality flags, local surface slopes <0° or >20°, and elevation differences exceeding ±200 m relative to reference DEMs were excluded. These filtering steps ensure robust and reliable elevation estimates suitable for subsequent glacier mass-change analysis.

(2) ASTER imagery data

The Advanced Spaceborne Thermal Emission and Reflection Radiometer (ASTER) is a key instrument onboard the Terra satellite, designed to acquire high-resolution multispectral imagery with a nominal repeat cycle of 16 days across 14 spectral bands [22]. The instrument includes three 15 m VNIR bands, six SWIR bands at 30 m resolution, and five TIR bands at 90 m resolution. ASTER also provides along-track near-simultaneous stereo image pairs with a temporal separation of ~55 s, enabling the generation of digital elevation models (DEMs). In the Karakoram region, the quality of ASTER data is affected by clouds, snow, and other factors, which reduce the quality of DEM. Therefore, we first only retained scenes with a cloud cover fraction of <10%. To minimize seasonal snow depth bias, we prefer to select DEMs acquired from the late ablation season (August–October) when the glacier surface is maximally exposed. Subsequently, DEMs exhibiting low stereoscopic coherence or failing to converge during co-registration were aggressively discarded. Finally, manual visual verification was conducted. A total of 7575 ASTER Level-1A images acquired between 2000 and 2022 were processed and analyzed.

(3) NASADEM

NASADEM was released in February 2020 and is distributed by the NASA Earth Observing System Data and Information System (EOSDIS) Land Processes Distributed Active Archive Center (LP DAAC). It is derived from a comprehensive reprocessing of the Shuttle Radar Topography Mission (SRTM) data using an improved interferometric algorithm [23]. In this reprocessing, ICESat laser altimetry data were incorporated as vertical control points to enhance elevation accuracy, while ASTER GDEM and additional auxiliary datasets were used to fill data voids.

NASADEM is provided at a spatial resolution of 1 arc-second (~30 m) and includes not only elevation data but also derived products such as slope, aspect, curvature, and a water body mask. The dataset is released as a tiled binary product and is freely available through NASA EOSDIS.

(4) Other datasets

Glacier inventory used in this study was obtained from the Randolph Glacier Inventory (RGI), which originated from the Global Land Ice Measurements from Space (GLIMS) initiative launched in 1999 as a joint effort by NASA and the U.S. Geological Survey (USGS) [24]. The RGI primarily derives glacier boundaries from Landsat and ASTER imagery using semi-automated delineation techniques [25]. In this study, glacier extents from RGI version 7.0 were employed to define glacierized areas for elevation and mass-change analysis.

To investigate the climatic drivers influencing glacier mass balance, monthly aggregated ERA5-Land reanalysis data were used, including near-surface air temperature and total precipitation [26]. Although the resolution is approximately 9 km, the temperature and rainfall data are better than similar data products in the Qinghai Tibet Plateau region [27,28]. A detailed summary of the datasets used in this study is presented in Table 1.

**Table 1:** Specific information of datasets used in this paper.

Category	Data	Time range	Resolution	Usage	Data source
Satellite altimetry	ICESat-2	2019-2024	20 m	Elevation calculation	https://nsidc.org/data/atl06/versions/6
Optical images	ASTER stereo	2000-2022	15 m		https://search.earthdata.nasa.gov/
DEM	NASA-DEM	2000	30 m	Reference DEM	doi.org/10.5067/MEaSUREs/NASADEM/NASADEM_HGT.001
Other datasets	RGI 7.0	--	--	Reference Glacier Range	https://daacdata.apps.nsidc.org/pub/DATASETS/nsidc0770_rgi_v7/
	ERA5	2000-2024	0.1 °	Analysis of influencing factors	https://www.ecmwf.int/

### 3.2. Methods

The technical workflow is illustrated in Fig 2. First, we preprocessed ASTER stereo imagery and satellite altimetry data, followed by co-registration with the NASADEM. Subsequently, glacier elevation changes over different periods were derived from the DEM differencing method. The annual and seasonal elevation changes were converted into mass balance estimates by applying a standardized density parameter. Finally, the spatio-temporal heterogeneity of glacier mass balance across the Karakoram region was analyzed by integrating meteorological and topographic data.

**Fig 2:**
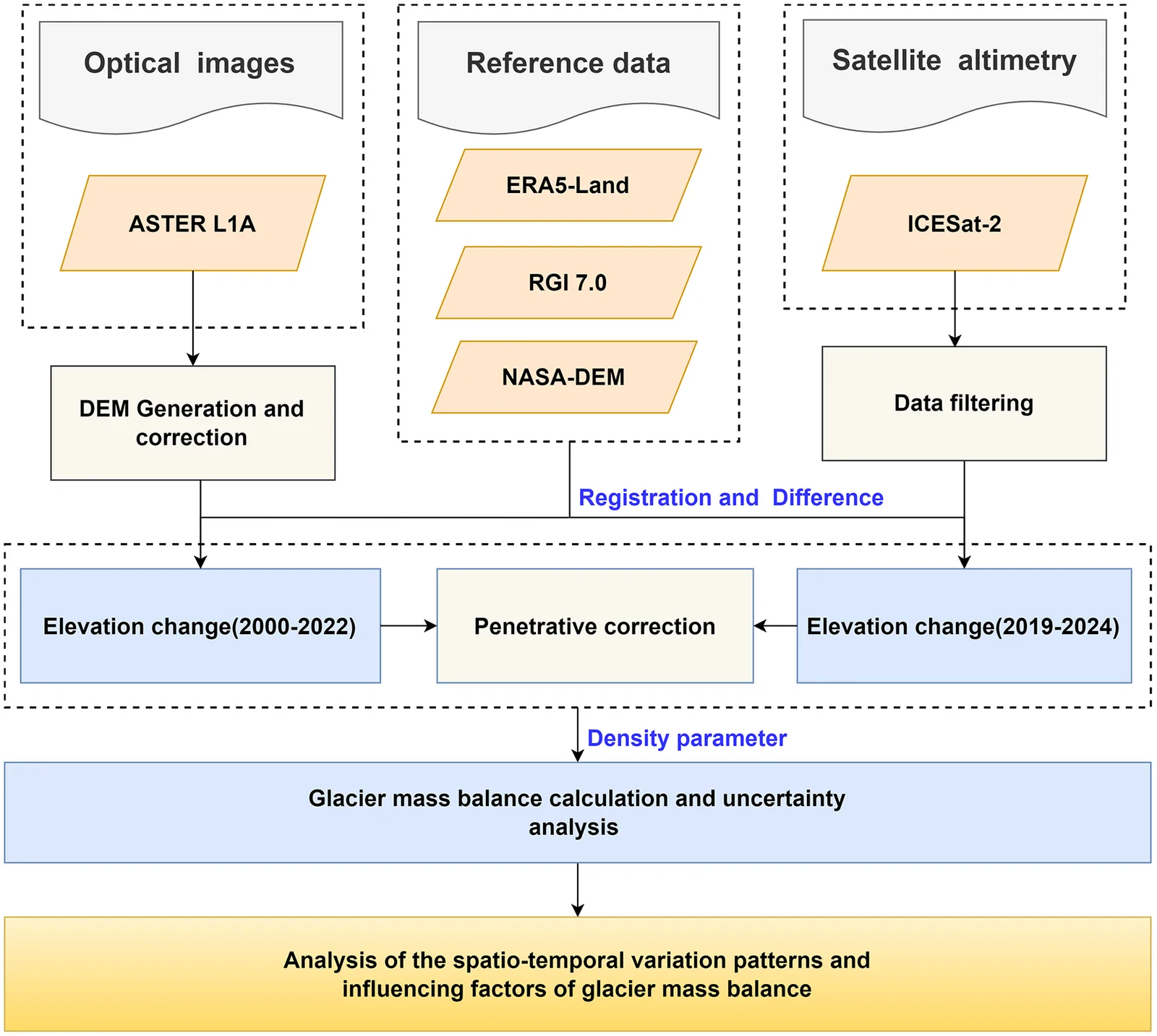
Technical flowchart of glacier mass balance analysis in the Karakoram based on ASTER imagery and ICESat-2.

#### 3.2.1 Data preprocessing.

To ensure consistency among the multi-source datasets, all ASTER, NASADEM, and ICESat-2 elevation datasets were transformed into a consistent horizontal coordinate reference system based on WGS84, with elevations referenced to the WGS84 ellipsoid, prior to further analysis.

ASTER Level-1A Version 003 imagery covering the Karakoram region from 2000 to 2022 was acquired for DEM generation. To satisfy stereo reconstruction requirements, scenes lacking either the nadir-looking (3N) or backward-looking (3B) bands were excluded, and only image pairs with complete along-track stereo observations were retained. ASTER DEMs were generated using the MicMac ASTER (MMASTER) processing chain [29], which applies optical stereo image matching under geometric constraints to produce DEMs at a spatial resolution of 30 m. Consecutive scenes acquired along the same orbital track were subsequently mosaicked to generate strip DEMs.

**To correct for relative spatial misalignments between multi-source datasets, all strip DEMs were systematically co-registered to the NASADEM reference surface following the analytical co-registration framework described below.** The co-registration was performed over stable, non-glacierized bedrock areas, and DEMs with a root mean square error (RMSE) exceeding 20 m were discarded. To eliminate severe data noise and terrain artifacts, the stable area was strictly restricted to vertical offsets of |dh| < 200 m and slopes of 0.1°–40°. The 3D translation vectors (dX, dY, dZ) were then resolved iteratively using a dynamic 3NMAD outlier filter. The refinement loop terminated when the incremental translation dropped below 0.02 m or reached a maximum of 30 iterations. To further mitigate remaining systematic biases, the pymmaster tool was applied to conduct differential fitting between DEM elevations and reference elevations over stable terrain, and the resulting bias terms were removed.

ICESat-2 Level-3A land ice elevation data (ATL06) were used as point-based glacier surface elevation observations, covering the period from December 2018 to August 2024. The data were accessed using the icepyx Python library in combination with the OpenAltimetry Application Programming Interface (API). Data queries were constrained to the bounding box of the study area DEM in the WGS84 coordinate system. Trajectory-based filtering was applied to ensure spatial consistency between ICESat-2 observations and the study domain, while maintaining a unified spatial reference and vertical datum.

Quality control procedures were applied to the ICESat-2 point cloud data prior to analysis. First, ATL06 elevation points were spatially matched with the corresponding DEM pixels, and elevation differences were computed. Observations with absolute elevation differences exceeding ±200 m were excluded to remove potential cloud-contaminated or spurious returns [30]. Surface slope was then derived from NASA DEM slope raster, and points located on slopes <0° or >20° were removed to reduce the influence of complex terrain on elevation accuracy. Finally, only ATL06 observations with a quality flag of 0 were retained, ensuring that only the highest-quality measurements were used in subsequent glacier elevation and mass-change analyses.

#### 3.2.2 Multi-Source DEM co-registration.

DEMs derived from different sources often exhibit spatial offsets, with elevation differences showing systematic dependencies on terrain slope and aspect [31]. To correct for such spatial misalignments, this study applies a DEM co-registration method based on the slope–elevation difference relationship [31]. Specifically, for a dependent DEM that requires co-registration to a reference DEM, the magnitude of the horizontal displacement vector, a, is assumed to be related to the elevation difference, dh, and the local surface slope, α, as expressed in Equation (1).


$$\begin{array}{c} \text{tan}(\alpha )=\frac{dh}{a} \end{array}$$ (1)

In addition, the elevation difference exhibits a directional dependence, with positive values on east-facing slopes and negative values on west-facing slopes. The orientation of the horizontal offset vector can be modeled by the cosine of the angle (Ψ) between the vector and the local slope aspect. This modeling establishes an analytical relationship between the individual elevation difference and the total elevation bias ($\overset{―}{dh}$).


$$\begin{array}{c} dh=a*\text{cos}(b-\Psi )*\text{tan}(\alpha )+\overset{―}{dh} \end{array}$$ (2)

To remove slope-dependent errors resulting from horizontal offsets, the vertical deviations are normalized, such that the relationship between elevation differences and slope aspect exhibits a clear sinusoidal pattern (Equations 3–4).


$$\begin{array}{c} \frac{dh}{\text{tan}(\alpha )}=a*\text{cos}(b-\Psi )*\text{tan}(\alpha )+c \end{array}$$ (3)

$$\begin{array}{c} =\frac{\overset{―}{dh}}{\text{tan}(\alpha )} \end{array}$$ (4)

By fitting the resulting scatter plot, the parameters a, b, and ccan be determined, which are subsequently used to calculate the horizontal offsets of the multi-source DEMs in the Xand Ydirections (Equations 5–6).


$$\begin{array}{c} X=a*\text{sin}(b) \end{array}$$ (5)

$$\begin{array}{c} Y=a*\text{cos}(b) \end{array}$$ (6)

#### 3.2.3 Penetration correction.

The penetration of imaging radar in snow- and ice-covered terrain is strongly influenced by the radar wavelength and the dielectric properties of surface materials. C-band radar can penetrate dry snow to depths of 1–2 m, whereas X-band radar exhibits much lower penetration capabilities (dry snow < 6 cm; wet snow < 10 cm) [32]. To minimize penetration-induced errors in SRTM DEMs over vegetated and snow-covered glacier areas, we applied a regionally validated penetration depth dataset published by Li et al. [33], which was derived by contrasting the SRTM C-band and X-band data over stable bedrock areas. This repository provides spatially explicit C-band penetration corrections and their corresponding uncertainty fields for each 1° × 1° grid cell.

#### 3.2.4 Glacier mass balance calculation.

To estimate glacier volume change, each sub-region was divided into multiple elevation bands with 50 m intervals. The mean elevation change, $E_{i}$, was calculated for each elevation band. The volume change for each band was then obtained by multiplying $E_{i}$ by the corresponding glacier area, $A_{i}$. Mass change was computed by applying a representative ice density of 850 ± 60 kg m ^−^ ³, following Huss [34], which approximates the typical mixture of glacier ice and snow for large-scale mass-balance assessments. The final glacier mass balance, dB, was calculated as follows:


$$\begin{array}{c} dB=\frac{\rho_{\text{ice}}}{\rho_{\text{water}}{\text{A}}_{\text{total}}}\sum_{i=\text{1}}^{n}A_{i}E_{i} \end{array}$$ (7)

In this study, the overall glacier mass balance was calculated using the conventional two-epoch geodetic method, in which glacier elevation changes were obtained from the difference between the reference DEM and the ICESat-2 elevation surface or ASTER DEM, divided by the corresponding time interval. This approach has been widely adopted in regional glacier mass balance studies.

#### 3.2.5 Uncertainty analysis.

The uncertainties in glacier mass balance estimates mainly arise from four factors: residual errors in DEM co-registration, SRTM/NASADEM radar penetration correction errors, glacier area uncertainties, and ice density variability. First, the residual error was estimated from the elevation differences between ICESat-2 observations and NASADEM over non-glacierized terrain. The corresponding elevation error, $\sigma_{g}$, was calculated using a 2 km decorrelation distance derived from the semi-variogram analysis [35]. For the ASTER DEM– NASA DEM differencing approach, the residual elevation error was estimated from the elevation differences over stable, non-glacierized terrain after DEM co-registration, using the resulting residual differences to characterize the uncertainty of the DEM-derived elevation changes. The penetration correction error, $\sigma_{\text{penetration}}$, was quantified as the standard deviation of the radar penetration depth within each elevation band below 6500 m, together with the penetration depth of non-glacier pixels [36]. These two components collectively define the error associated with glacier surface elevation measurements.


$$\begin{array}{c} \sigma_{\text{h}}=\sqrt{\sigma_{\text{g}}^{\text{2}}+\sigma_{\text{penetration}}^{\text{2}}} \end{array}$$ (8)

By further incorporating uncertainties associated with glacier area (±10%) and ice density (±60 kg m ^−^ ³), the overall volume uncertainty, $\sigma_{V}$, and mass uncertainty, $\sigma_{B}$, were calculated as follows:


$$\begin{array}{c} \sigma_{\text{V}}=\sqrt{{\left(\sigma_{h}A\right)}^{\text{2}}+{\left(\sigma_{A}dh\right)}^{\text{2}}} \end{array}$$ (9)

$$\begin{array}{c} \sigma_{\text{B}}=\sqrt{{\left(\frac{\rho_{\text{ice}}}{\rho_{\text{water}}A}\sigma_{V}\right)}^{\text{2}}+{\left(\frac{V}{\rho_{\text{water}}A}\rho_{\text{ice}}\right)}^{\text{2}}} \end{array}$$ (10)

## 4. Results and analysis

### 4.1. Glacier elevation changes and mass balance results from 2000 to 2024

#### 4.1.1 Spatial variation characteristics.

Fig 3 illustrates the spatio-temporal variations in glacier mass balance across the Karakoram region. The results indicate a general retreat of glaciers between 2000 and 2024, with a cumulative surface elevation loss exceeding 1 m. The average elevation change rate was −0.0454 ± 0.0049 m/a, with relatively small associated uncertainties. Assuming an ice density of 850 ± 60 kg m ^−^ ³ [34], the corresponding mass balance was estimated at −0.0386 ± 0.0032 m w.e. a ^−^ ¹.

**Fig 3:**
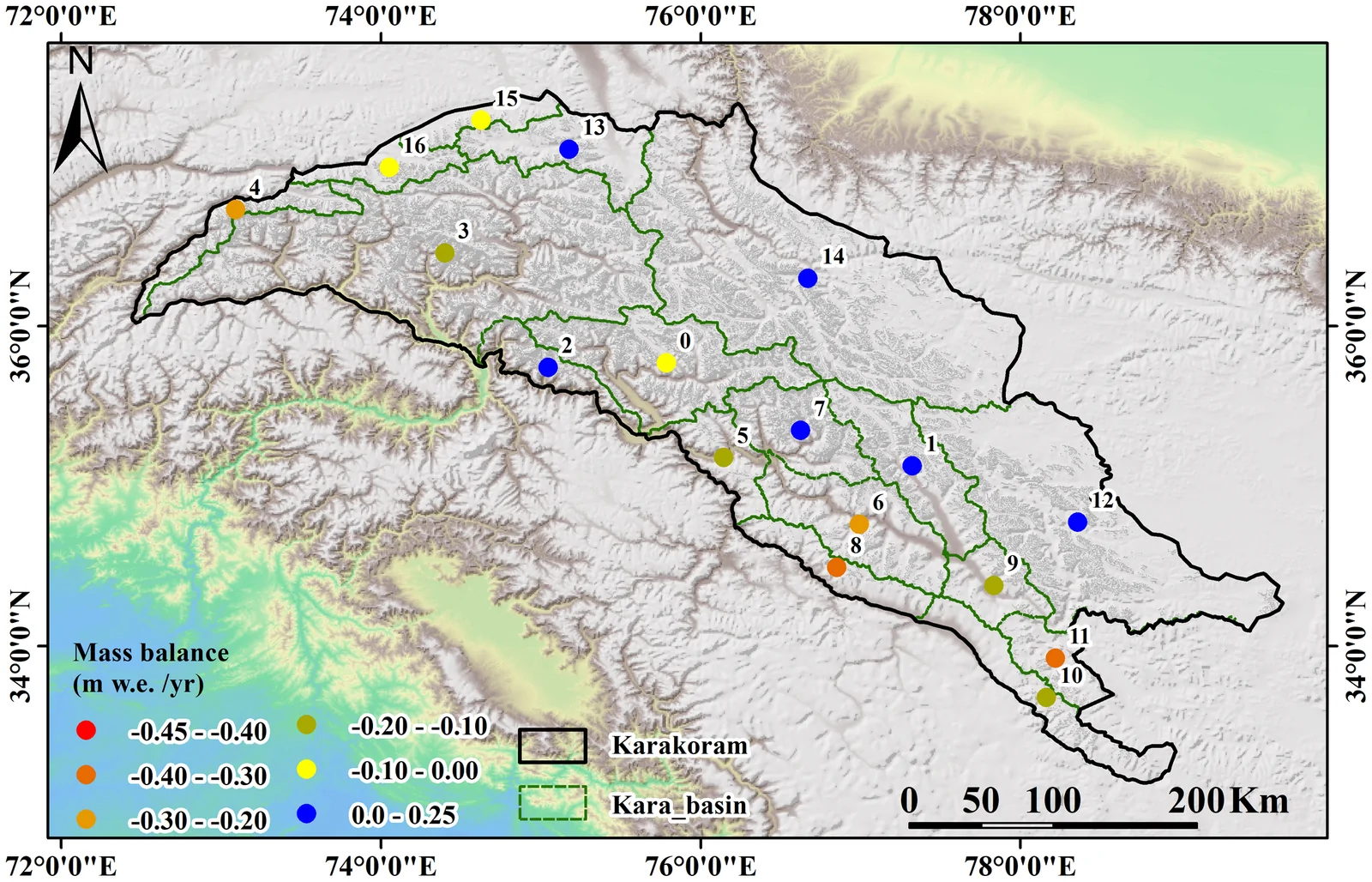
Spatial distribution of glacier mass balance in Karakoram. The basemap is derived from NASA DEM.

To examine spatial variations in greater detail, the Karakoram region was subdivided into 17 sub-basins based on the HydroATLAS database. Glacier mass balance varied substantially among the 17 sub-basins, with relatively positive or near-equilibrium conditions in the northern and central Karakoram but stronger mass loss in several southern and eastern basins. Specifically, basins 13, 14, and 12 in the northern and central Karakoram exhibited slight glacier accumulation, whereas basins 4, 6, 8, and 11 in the southern and eastern Karakoram experienced mass loss. Among all sub-basins, Basin 8 underwent the most pronounced glacier loss, with a mass balance of −0.35 ± 0.027 m w.e. a ^−^ ¹, while basin 7 showed the highest glacier gain, at 0.067 ± 0.006 m w.e. a ^−^ ¹.

#### 4.1.2 Temporal variation characteristics.

Fig 4 shows the evolution of glacier mass balance across the 17 sub-basins over five consecutive observation periods from 2000 to 2024. During 2000–2005, the region exhibited an overall near-equilibrium state characteristic of the classic anomaly: most basins showed mass balance rates close to zero, basin 5 in the central Karakoram maintained a positive mass balance (+0.2 to +0.4 m w.e. a ^−^ ¹), and only a few southern basins (e.g., 1, 11) experienced slight negative mass loss. From 2005 to 2010, the positive balance area gradually shrank, the positive signal in basin 5 disappeared, and most central and southern basins shifted to weak negative balance, indicating the initial weakening of the anomaly. During 2010–2014, almost all sub-basins shifted to negative mass balance, marking a transition from predominantly near-equilibrium conditions to widespread mass loss. During 2014–2019, glacier ablation further intensified, particularly in the southern Karakoram (basin 7, 8), where mass balance rates dropped to −0.2 to −0.4 m w.e. a ^−^ ¹, while northern basins (13, 14) remained relatively stable. The 2019–2024 period exhibited the most negative mass balance among the five observation periods, when the entire region entered a phase of accelerated ablation. The western Karakoram (basin 2, 3) experienced the most severe mass loss (> −0.4 m w.e. a ^−^ ¹), southern basins (5, 6, 8, 9) showed enhanced ablation, and only the northernmost basins maintained relatively mild negative balance. These results indicate a substantial weakening of the Karakoram Anomaly at the regional scale after 2020, although positive mass balance persisted in a limited number of northern and central basins.

**Fig 4:**
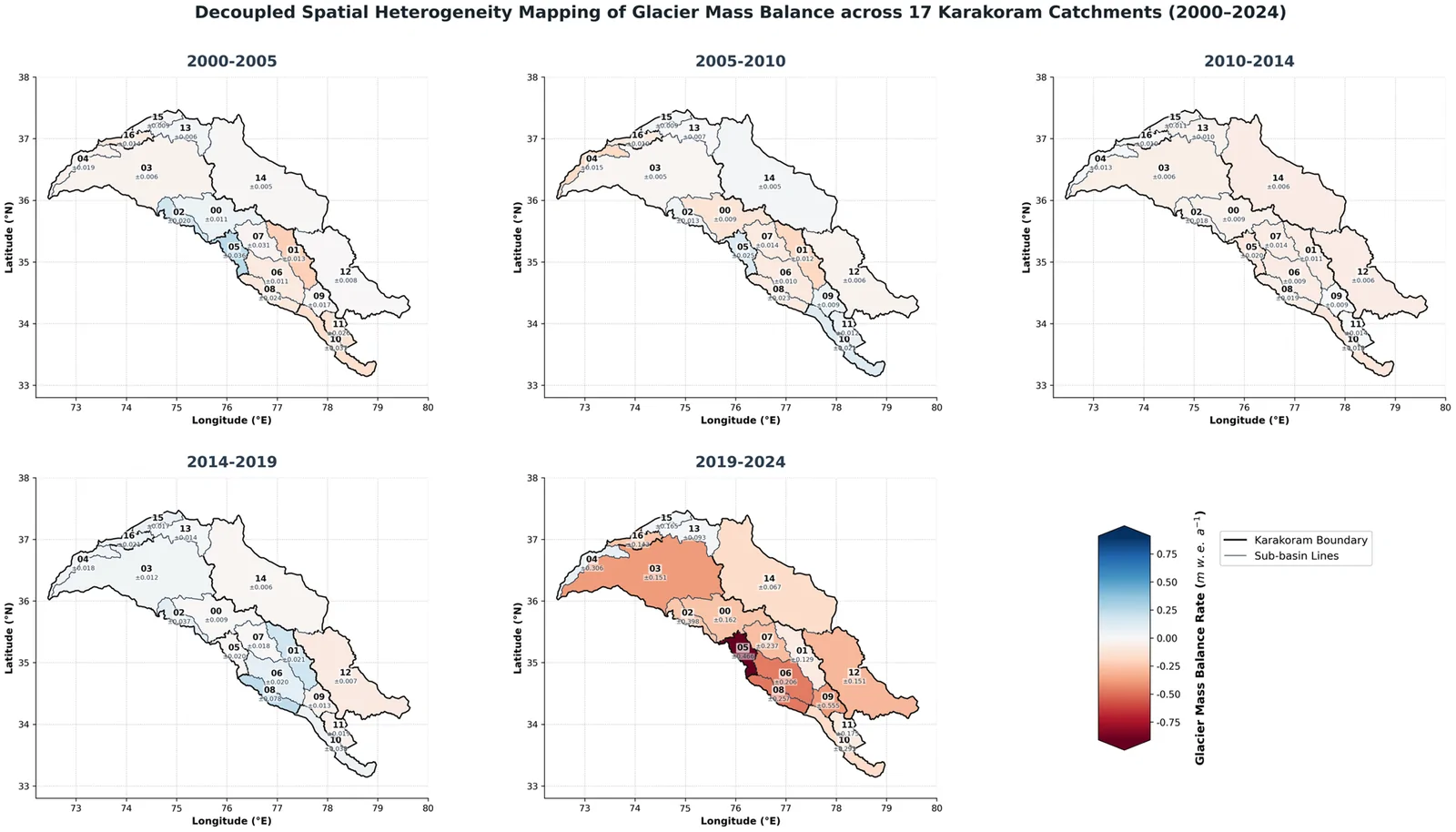
The spatiotemporal evolution of glacier mass balance across 17 Karakoram basins.

The mass balance of regional glaciers shows a clear four stage evolution (Fig 5): from 2000 to 2010, it was a weak negative stage (−0.02 ± 0.08 m w.e. a ^−^ ¹), gradually decreasing to −0.06 ± 0.07 m w.e. a ^−^ ¹ from 2010 to 2014, temporarily returning to a near equilibrium state from 2014 to 2019. Rapid glacier loss accelerated after 2019, and this pattern occurred across most basins, such as basins 2, 3, 5, and 6.

**Fig 5:**
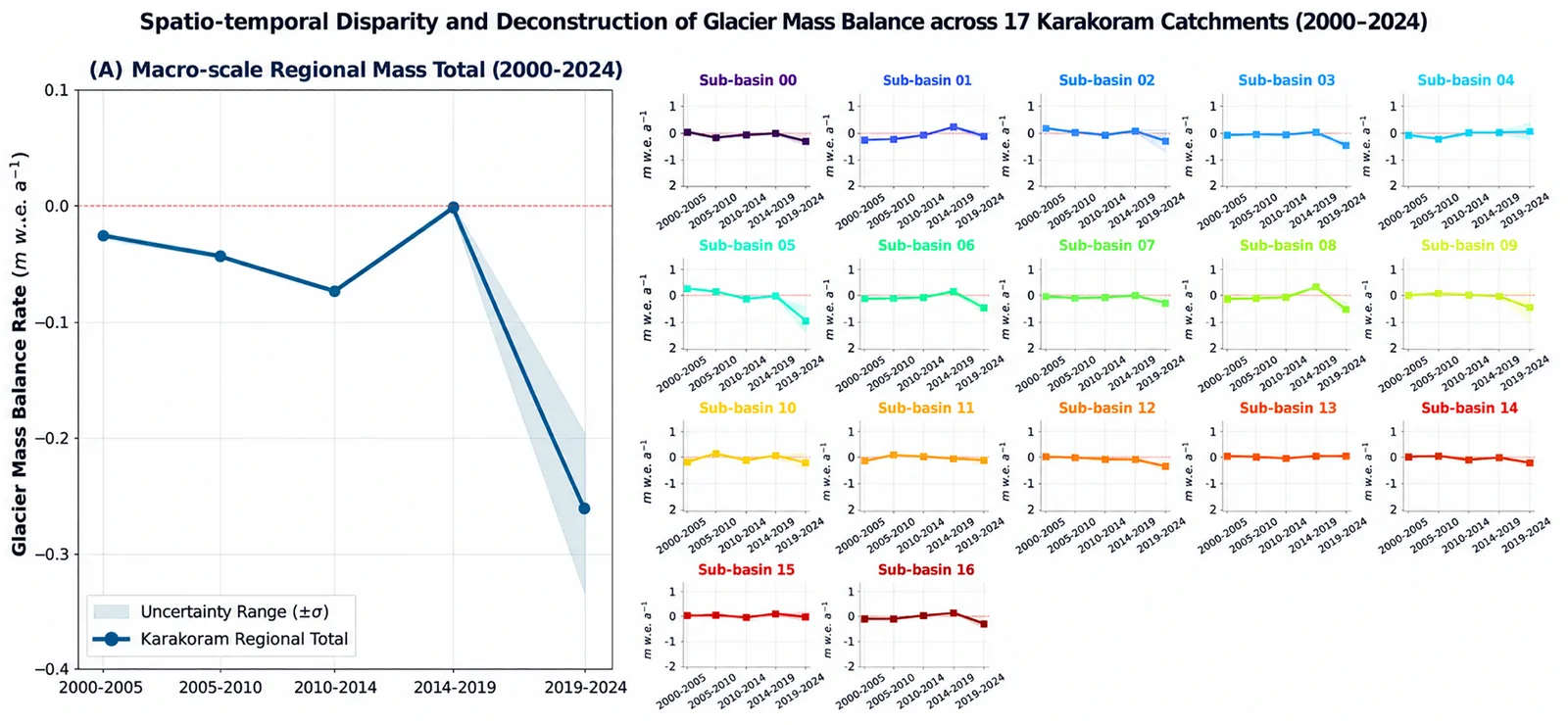
Temporal changes in glacier mass balance in entire region and each basin.

### 4.2. Seasonal variation characteristics of the Karakoram Glacier from 2019 to 2024

Using the first quarter of 2019 ICESat-2 data as a reference baseline, seasonal elevation changes were analyzed for each quarter from 2019 to 2024. The seasonal elevation changes were closely aligned with seasonal temperature variations, with elevation increases mainly occurring between March and May and decreases between August and October (Fig 6). During 2019–2021, Karakoram glaciers remained in a fluctuating near‑equilibrium state. This phase was characterized by alternating winter accumulation and summer ablation, with no sustained long‑term trend. After 2021, the magnitude of elevation gain during the accumulation season gradually diminished, while elevation loss during the retreat season intensified. Consequently, the imbalance between seasonal accumulation and ablation increased over time, leading to increasingly negative net elevation changes after 2021. The extreme melting event during the third quarter of 2022 represented the largest single-quarter mass loss during the study period, with a net loss of approximately 2.85 m w.e., accounting for about 26% of the cumulative negative mass change from 2019 Q1 to 2024 Q3.

**Fig 6:**
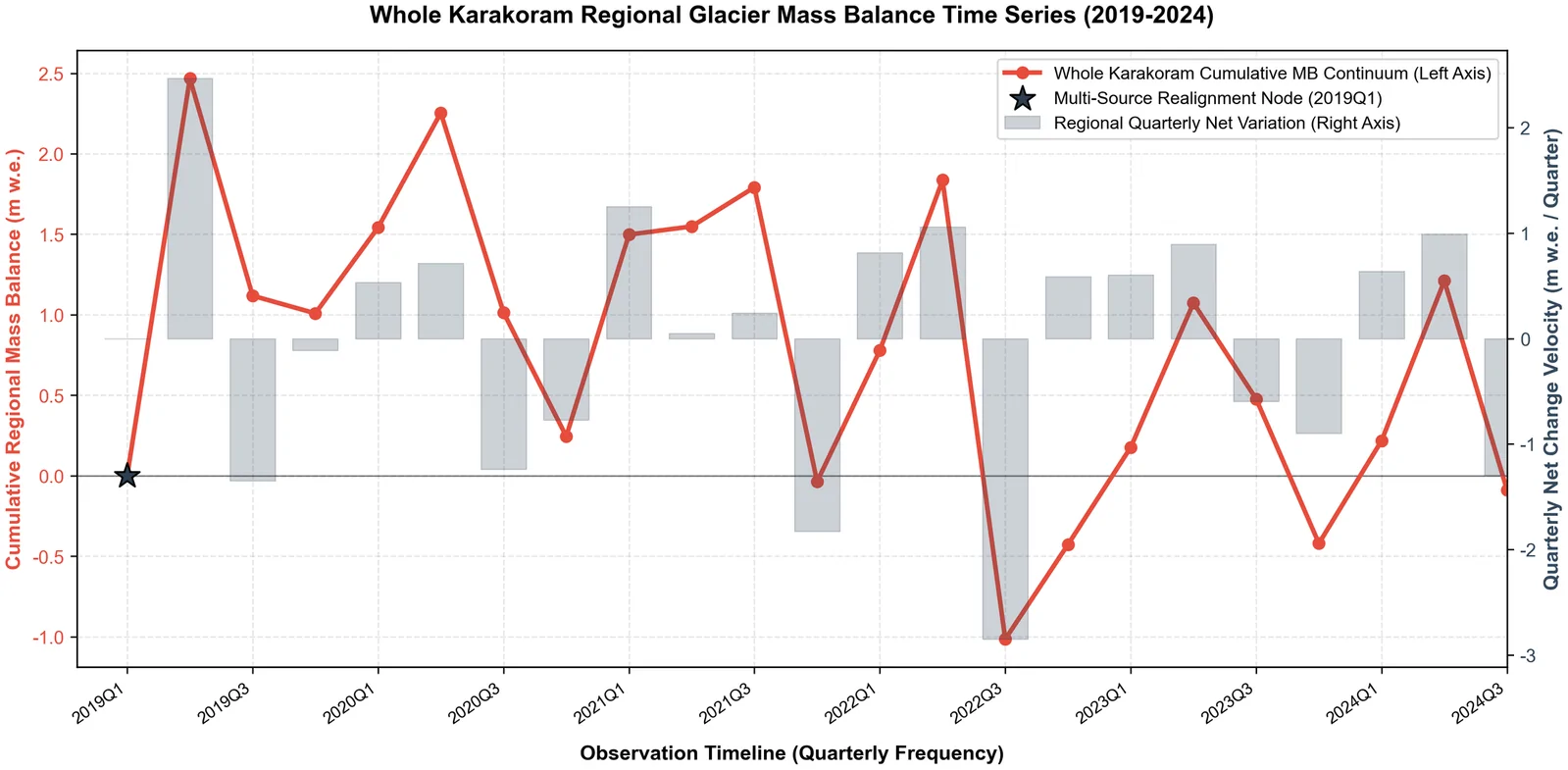
Seasonal variation trend of glacier mass balance in Karakoram region.

Considering inter-basin differences, the seasonal mass change for each of sub-basins (Basin IDs 0–16) was calculated. The results are presented as a heatmap (Fig 7), where each cell represents the mass balance change for a given basin during a specific quarter. The color scale on the right indicates the magnitude and direction of change: red denotes mass gain, blue denotes mass loss, and darker shades correspond to larger absolute values.

**Fig 7:**
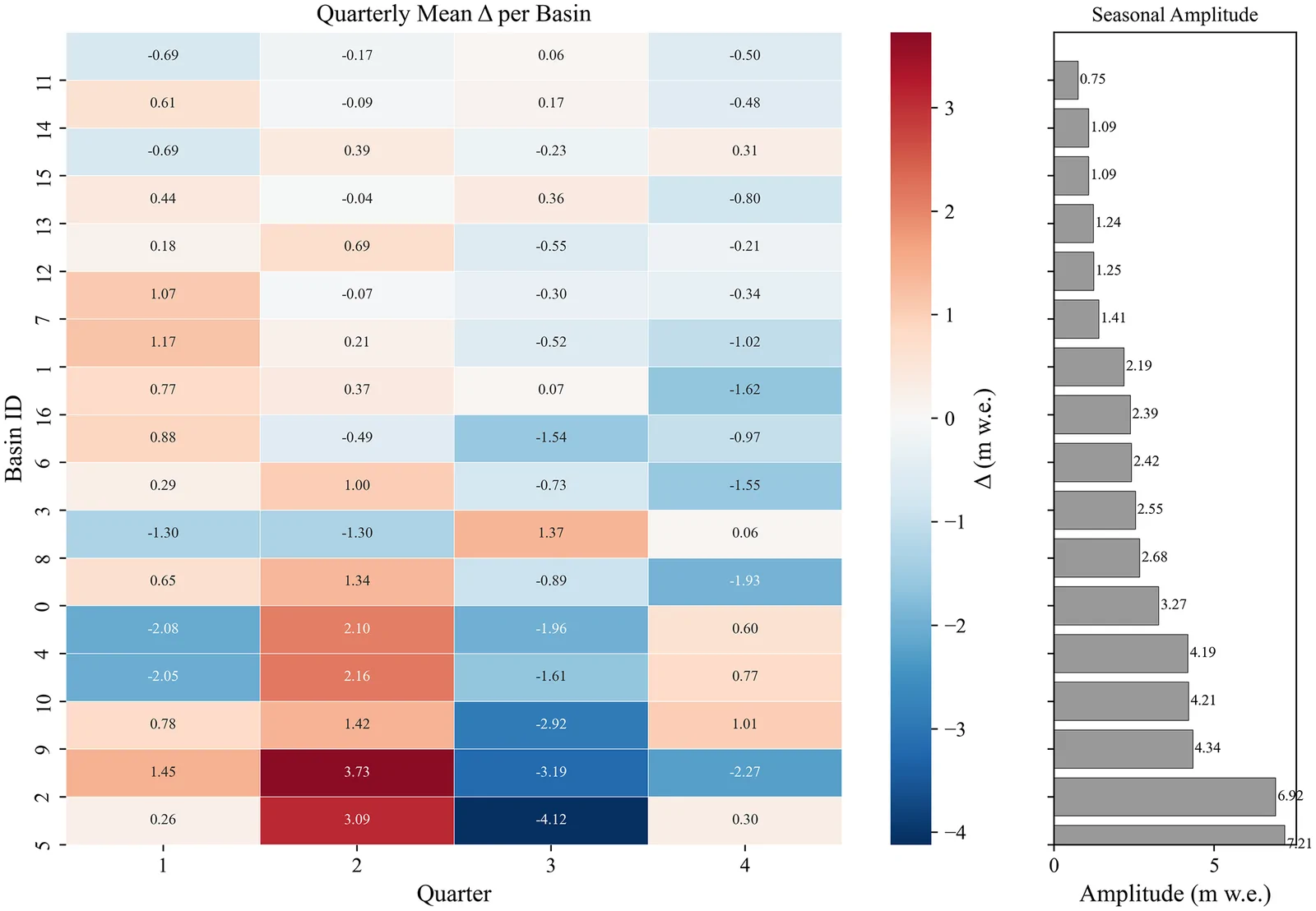
Average quarterly mass change of each basin over four quarters.

The analysis shows that most basins generally experience mass gains during the first and second quarters, likely associated with lower spring and winter temperatures favoring glacier accumulation. Conversely, mass loss predominates in the third and fourth quarters, consistent with higher summer temperatures enhancing retreat. Notably, there are pronounced differences in both the seasonal trends and the amplitude of mass changes among individual basins. For instance, basins 4 and 10 exhibited substantial mass loss even in the first quarter, unlike most other sub-basins. The largest seasonal amplitudes occurred in basins 2 and 5, which exhibited larger seasonal accumulation and ablation changes than most other sub-basins. In contrast, basins 1, 13, and 14 showed the smallest amplitude, likely attributable to relatively stable environmental and climatic conditions. Fig 8 shows the time series curves of mass balance for each basin, further visually demonstrating the differences in changes among different basins.

**Fig 8:**
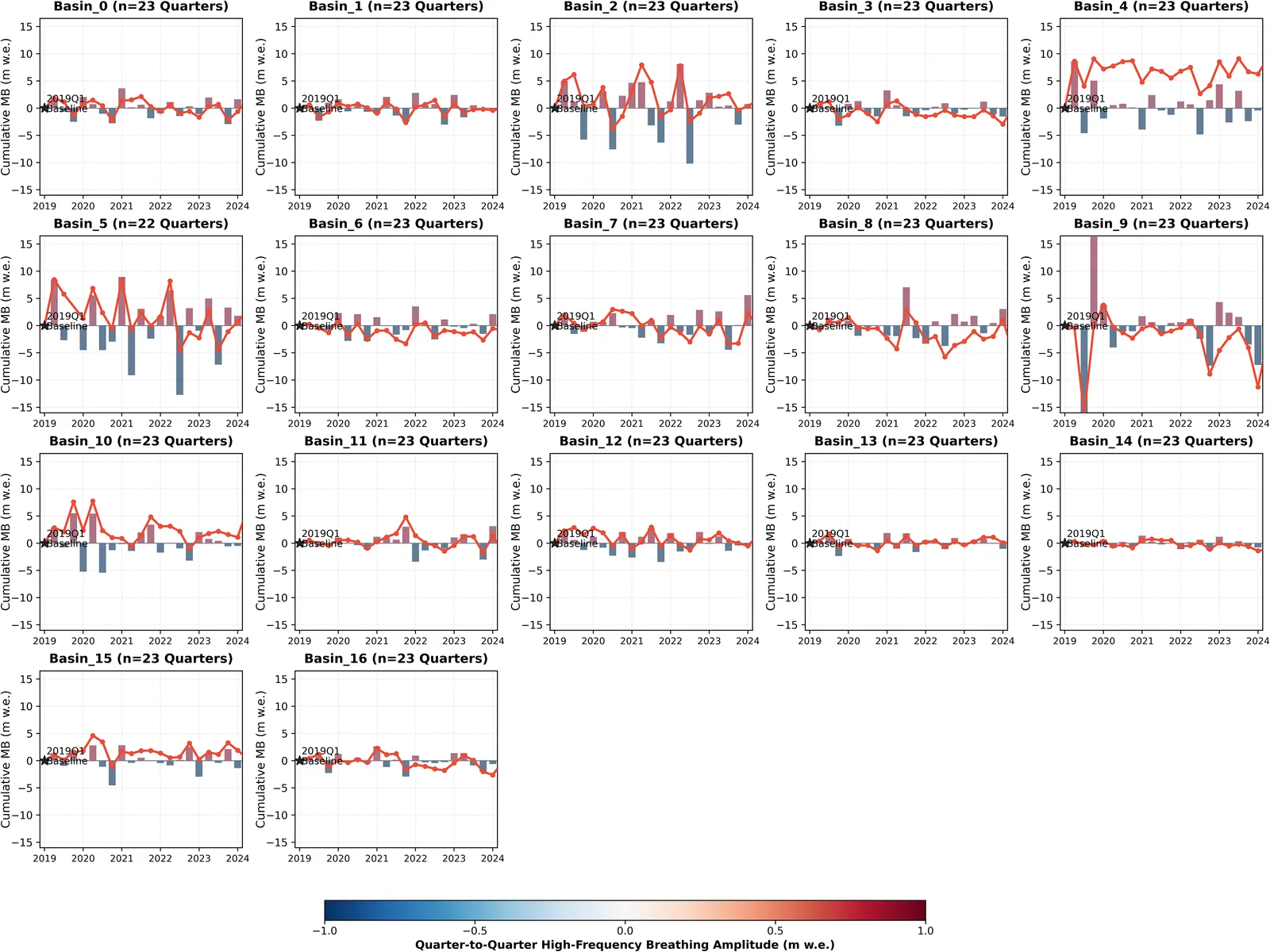
Seasonal variation trend of glacier mass balance in each sub-basin.

## 5. Discussion

### 5.1. Spatiotemporal variation characteristics of Karakoram glaciers and comparison with existing results

Previous studies have indicated that the Karakoram anomaly was first documented in the early 21st century and persisted until approximately 2010 [37]. Subsequently, glaciers in some areas began to retreat, with the mass balance exhibiting an overall negative trend [38–40], particularly in the eastern Karakoram [41,42].

In this study, glacier mass balance over the past 25 years was quantified across multiple spatial and temporal scales by combining ASTER DEM and ICESat-2 datasets. The results indicate that prior to 2010, glaciers across the Karakoram generally experienced accumulation, consistent with the previously reported Karakoram anomaly [37]. Post-2010, although the overall glacier state transitioned toward mass loss, the anomaly did not entirely vanish; instead, it exhibited distinct regional and temporal characteristics. Spatially, positive mass balance persisted primarily in the northern Karakoram (e.g., basins 12 and 14) and central regions (basins 1 and 7). Temporally, analysis of interannual and seasonal variations suggests a reappearance of the anomaly between 2018 and 2020. In contrast, the southeastern Karakoram (e.g., basins 6, 8, and 11) experienced continuous mass loss, indicating that the anomaly had largely disappeared in this area. After 2020, most basins showed net mass depletion, with only a few eastern basins retaining minor positive trends, suggesting that the regional expression of the anomaly had substantially weakened and become spatially restricted. Whether this anomaly will re-emerge in the future remains uncertain, underscoring the need for continued monitoring.

Table 2 summarizes current research findings on glacier mass balance in the Karakoram region. Despite differences in data sources, time spans, and methodologies, the mass balance results from Brun et al. [38], Shean et al. [40], Hugonnet et al. [2], and Wang et al. [43] are relatively consistent, all finding that the glaciers were in a slightly negative mass balance state (approximately −0.03 to −0.05 m w.e. a ^−^ ¹) before 2019. However, after 2020, Wang et al. [43] observed a significant acceleration in glacier melting, with the mass depletion rate from 2019 to 2022 (−0.30 ± 0.22 m w.e. a ^−^ ¹) being 10 times that of the rate from 2000 to 2019.

**Table 2:** Current research findings on glacier mass balance in the Karakoram region.

Study	Data	Time Range	Mass balance/ m w.e. a^−1^
Brun et al. [38]	ASTER DEM	2000-2016	−0.03 ± 0.07
Shean et al. [40]	ASTER DEM	2000-2018	−0.04 ± 0.04
Hugonnet et al. [2]	ASTER DEM	2000-2019	−0.05 ± 0.01
Fan et al. [39]	ICESat-2 and NASADEM	2000-2021	−0.03 ± 0.12
Wang et al. [43]	ICESat-2 and NASA DEM	2019-2022	−0.30 ± 0.22
		2000-2019	−0.03 ± 0.01
Zhou et al. [44]	ICESat-2 and NASA DEM	2000-2022	−0.04 ± 0.15
This study	ICESat-2 and NASA DEM	2000-2024	−0.0386 ± 0.0032
		2019-2024	−0.257 ± 0.074
	ASTER DEM	2000-2022	−0.073 ± 0.06

Compared to previous studies, this study extends the observation period through 2024. Our analysis reveals a mass loss rate of −0.0386 ± 0.0032 m w.e. a ^−^ ¹, which is in close agreement with Fan et al. [39], Zhou et al. [42], and Wang et al. [43]. Moreover, our calculated mass balance rate for the period 2019–2024 is also consistent with that of Wang et al. [43], which further corroborates the robustness and reliability of our results. The glacier mass balance during 2019–2024 (−0.257 ± 0.074 m w.e. a ^−^ ¹) was substantially more negative than the long-term average for 2000–2024, even when the associated propagated uncertainties are considered, indicating accelerated glacier mass loss during the recent period.

The discrepancies between our results and previous studies can largely be attributed to differences in data sources, spatio-temporal scope, topographic correction, and density parameters. First, the ASTER‑derived mass balance is more negative than the ICESat‑2 estimate for the overlapping period. This discrepancy likely stems from differences in spatial sampling: ASTER DEMs provide near‑complete coverage of the glacier surface, including high‑altitude accumulation zones that may have experienced thinning over the longer ASTER period, whereas ICESat‑2 footprints are potentially located in lower‑elevation, flatter areas that may have experienced milder changes. Additionally, the higher spatial resolution of ASTER DEMs may capture localized, intense ablation features that are missed by the sparse ICESat‑2 footprints. Consequently, the ASTER-derived mass loss rate (−0.073 ± 0.06) for 2000–2022 is markedly higher than the altimetry-derived rate.

Differences in temporal coverage contribute to the discrepancies among published estimates. Previous studies generally ended before 2020, whereas our analysis extends to 2024 and captures the recent acceleration of glacier mass loss. Given the strong temporal variability of glacier change in the Karakoram, different observation periods inevitably lead to different long-term mean mass balances. In addition, differences in glacier inventories and spatial coverage may also affect regional estimates. Despite these differences, both methods consistently indicate a shift toward net mass loss in the Karakoram after 2010.

Then, methodological differences in DEM co-registration may introduce systematic biases, particularly in steep terrain where small horizontal offsets can be amplified into large elevation errors. Moreover, different studies adopted different radar penetration correction schemes. While some studies applied uniform correction values, this study employed spatially distributed penetration estimates derived from the SRTM C-band and X-band difference, which may reduce regional biases, especially in high-elevation accumulation zones. Finally, although most studies used the same density parameters, variations in the handling of density uncertainty and error propagation may still lead to differences in the reported uncertainty limits.

### 5.2. Impacts of climate factor on glacier mass balance

Substantial evidence underscores climate change as the principal driver of glacier dynamics, wherein temperature and precipitation collectively govern glacial mass balance by modulating surface energy and mass budgets [30,45–47]. To quantify the climatic factors on mass balance, annual and summer temperature and rainfall series in the Karakoram region from 2000 to 2024 were collected. The climate record shows a concurrent increase in temperature and precipitation across the Karakoram, but the magnitude of change was substantially greater during summer. Mean annual temperature increased at 0.0057 °C a ^−^ ¹ and annual precipitation at 1.7 cm a ^−^ ¹, whereas the corresponding summer trends reached 0.0123 °C a ^−^ ¹ (Fig 9a) and 2.18 cm a ^−^ ¹ (Fig 9b), respectively. The accelerated summer warming directly intensifies melt, leading to a contraction of the accumulation area and an expansion of the retreat zone, thereby impeding glacier mass gain. Although summer precipitation has increased, higher temperatures promote a phase shift from solid to liquid precipitation. Liquid precipitation not only fails to contribute effectively to mass accumulation but also exacerbates ice melt by lowering surface albedo and releasing latent heat.

**Fig 9:**
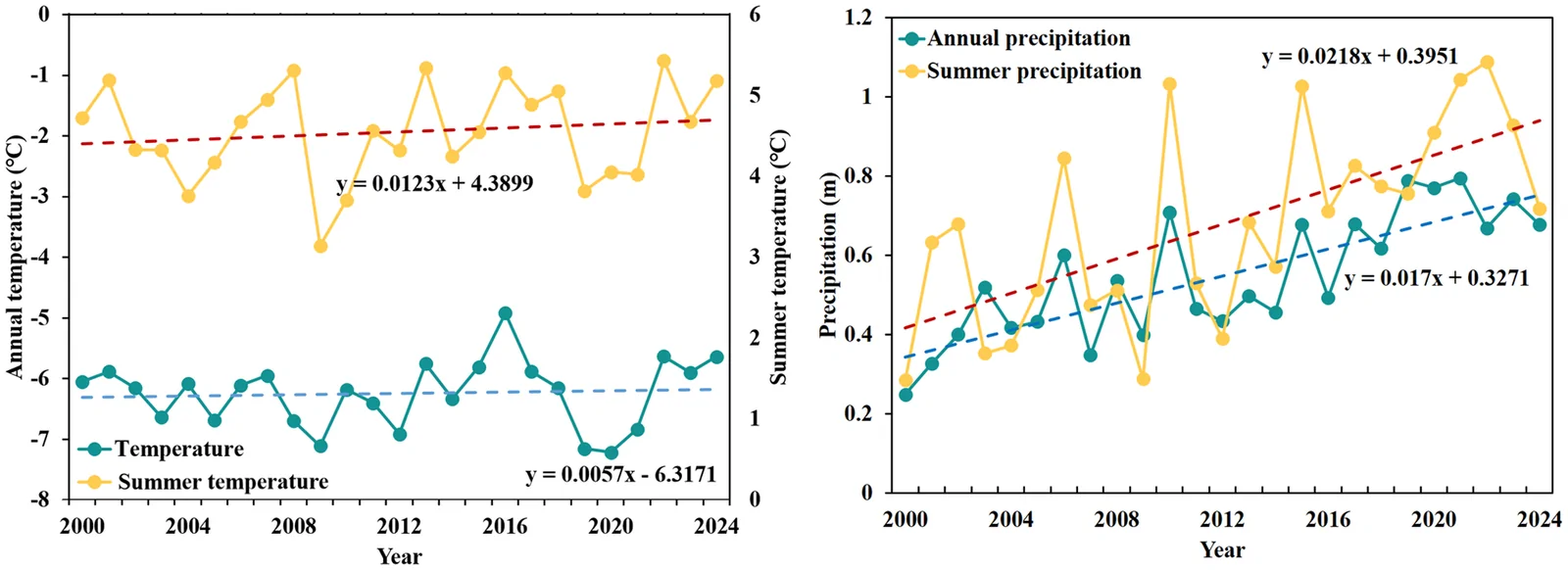
Variation of temperature (a) and precipitation (b) in the Karakoram region from 2000 to 2024.

The phased climate anomalies provide further context for the temporal evolution of the Karakoram Anomaly (Table 3). Relative to the 2000–2024 mean, summer temperature was 0.09 °C lower during 2000–2010, increased to 0.03 °C above the reference during 2010–2020, and reached 0.12 °C above the reference during 2020–2024. This obvious warming may be related to recent extreme heat waves and compound heatwaves [48], contributing to the widespread dissipation of the Karakoram anomaly [44].

**Table 3:** Temperature and rainfall anomalies in the Karakoram in different time periods.

Time period	Temperature anomaly	Precipitation anomaly	Karakoram anomaly	Glacier Mass Balance
2000-2010	−0.09 °C	−0.15 m	Overall existence	Overall stable or accumulating state
2010-2020	0.03 ℃	0.06 m	Regional and phased existence	Overall losses, some sectors stable
2020-2024	0.12 ℃	0.26 m	Only a few areas remained	Overall losses, a few sectors stable

Over the same periods, precipitation changed from 0.15 m below the long-term mean to 0.06 m above it and subsequently to 0.26 m above it. The transition from a relatively cool and dry early period to a warmer and wetter recent period coincided with the shift from predominantly stable or positive mass balance to widespread mass loss. This temporal correspondence supports an important role for the recent warming in the weakening of the Karakoram Anomaly, although the available data do not allow the individual contributions of temperature and precipitation to be separated quantitatively at the regional scale.

Spatially, correlating long‑term mass balance rates (2000–2024) with mean climatic conditions across individual glacier basins revealed weak negative correlations between mass loss and both basin‑averaged temperature and precipitation (Fig 10). To evaluate the assumption of spatial independence among the 17 sub-basins, Moran’s I statistics were calculated for basin-scale mass balance and climate variables. No significant spatial autocorrelation was detected for glacier mass balance (Moran’s I = 0.119, p = 0.184) or mean temperature (Moran’s I = 0.041, p = 0.449), whereas mean precipitation exhibited significant positive spatial autocorrelation (Moran’s I = 0.313, p = 0.006). Therefore, the Pearson correlation involving precipitation should be interpreted as a regional association rather than evidence of a spatially independent causal relationship.

**Fig 10:**
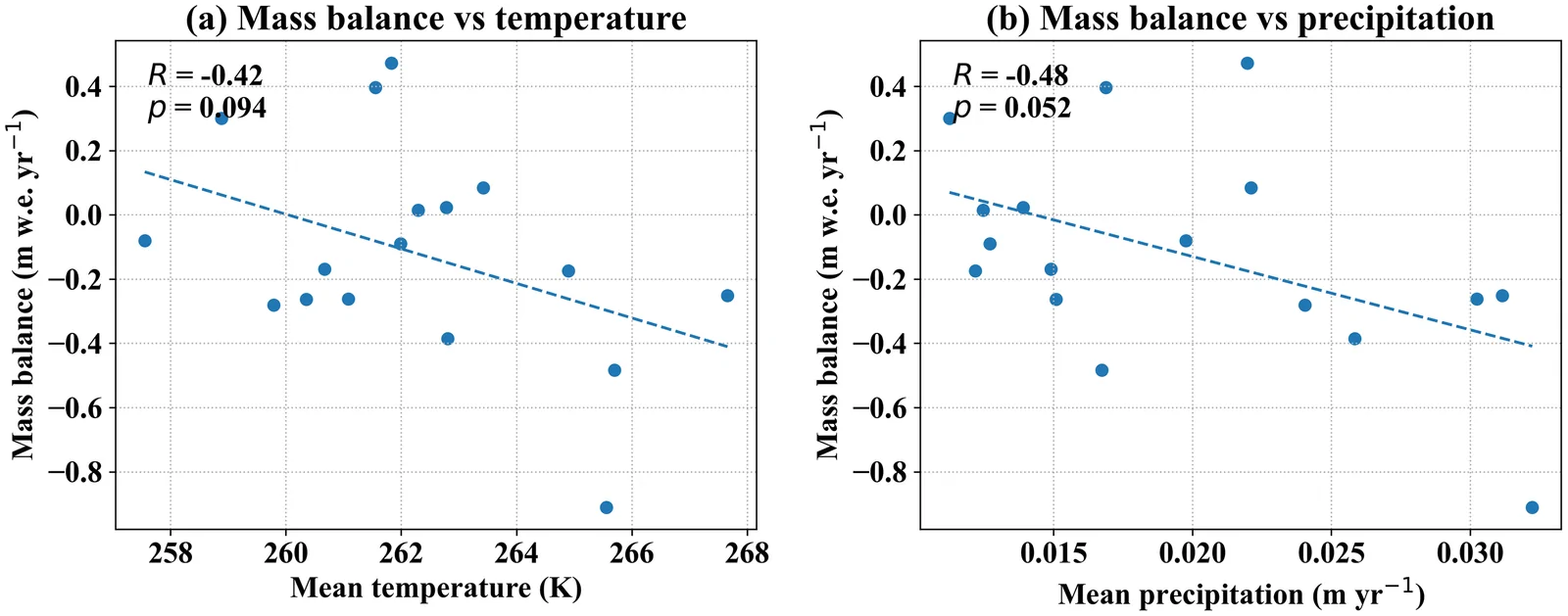
Correlation analysis of glacier mass balance with (a) temperature, and (b) precipitation.

Although ERA5-Land provides one of the highest-resolution global reanalysis datasets currently available (~9 km), its spatial resolution remains insufficient to fully resolve the pronounced topographic complexity of the Karakoram, where elevation differences commonly exceed 5,000 m within individual grid cells [49]. Consequently, local topoclimatic processes, including valley circulations, glacier-induced katabatic winds, and terrain-related radiative effects, are inevitably smoothed in the reanalysis, potentially affecting glacier-scale climate forcing. Previous evaluations in the Himalaya have similarly shown that ERA5-Land performs well at representing regional climatic variability but cannot adequately reproduce local atmospheric processes without further statistical or dynamical downscaling [49]. Therefore, the relationships between glacier mass balance and climatic variables identified in this study should be interpreted as regional-scale associations rather than glacier-specific climatic responses. Future work integrating high-resolution regional climate models and field observations will help better resolve these local processes.

### 5.3. Impacts of topographic factor on glacier mass balance

In addition to the special role of climate, topographic characteristics represent important local factors driving spatial heterogeneity in glacier mass balance across basins [50,51]. In this study, mean elevation shows a positive relationship with glacier mass balance across the 17 sub-basins (Fig 11a), indicating that higher-elevation basins generally experienced less negative or more positive mass-balance conditions. This pattern is consistent with the lower thermal conditions expected at higher elevations, which can reduce melt intensity and favor the retention of solid precipitation. Conversely, lower-elevation basins are more exposed to above-freezing temperatures during the ablation season and therefore tend to experience greater mass loss. The elevation relationship is therefore consistent with the spatial contrast observed in glacier mass balance across the Karakoram.

**Fig 11:**
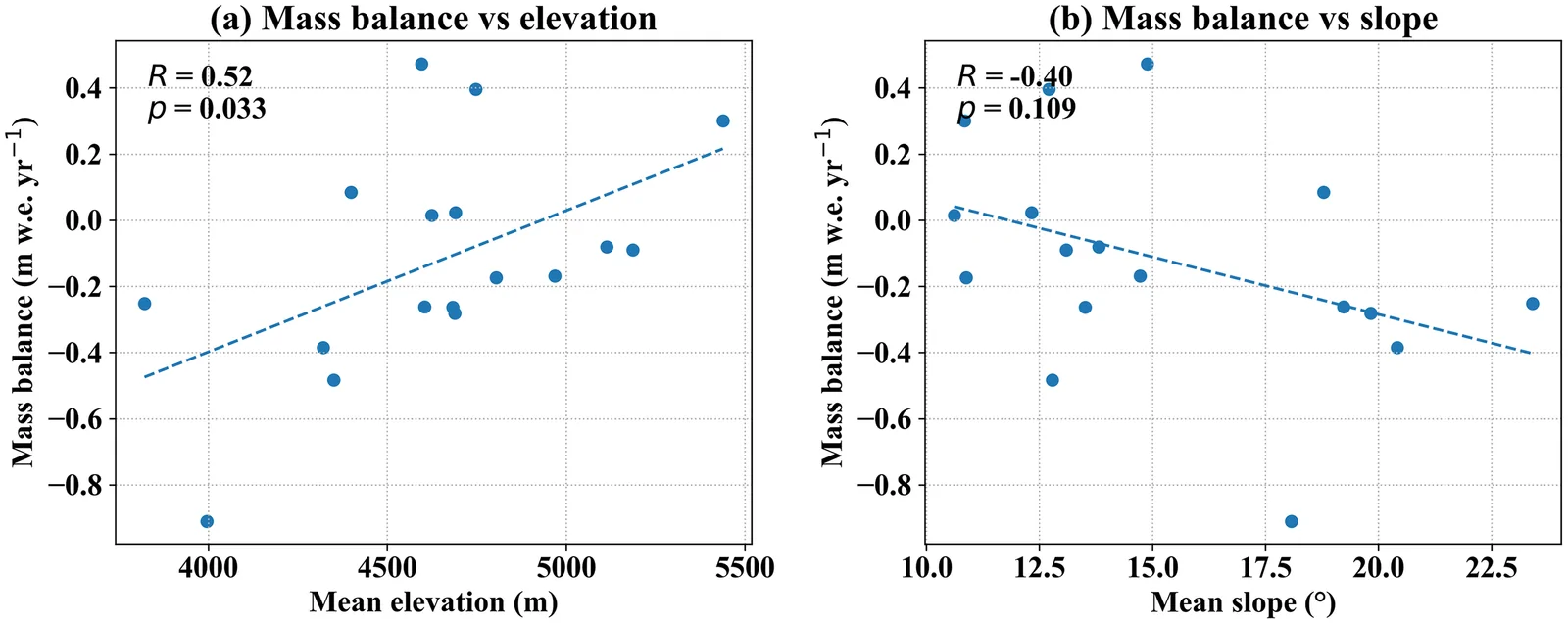
Correlation analysis of glacier mass balance with (a) elevation, and (b) slope angle.

In contrast, the relationship between mean slope and mass balance is weak and statistically non-significant (R = −0.40, p = 0.109). The present dataset therefore does not provide sufficient evidence for an independent slope effect at the basin scale. The weak negative tendency may reflect the limited number of sub-basins (n = 17), as well as the influence of factors not explicitly considered here, such as debris cover and glacier hypsometry. This result also indicates that elevation alone cannot explain the full spatial heterogeneity of mass balance, supporting the need to consider climatic conditions and glacier-specific characteristics together.

### 5.4. Uncertainty from glacier surge activity

The presence of surge‑type glaciers in the Karakoram introduces considerable uncertainty when assessing the persistence of the Karakoram Anomaly. During their active phase, these glaciers rapidly advance and cause localized surface thickening, thereby making a positive contribution to the regional mass balance signal. This dynamic thickening may be mistaken for climate‑driven stability or even mass gain, thus exaggerating the apparent strength and duration of the anomaly. Conversely, when surge‑type glaciers enter a quiescent phase, they may thin or retreat, potentially accelerating the perceived mass loss. According to the latest surge‑type glacier inventory [52], there are 454 surge‑type glaciers in the Karakoram region since 2000, accounting for approximately 3.5% of the total number of glaciers. This relatively low areal coverage suggests that the influence of glacier surges on the regional average mass balance may be inherently limited. To further quantify this influence, we analyzed surface elevation changes for a subset of 100 surge‑type glaciers using our ASTER DEM dataset (Fig 12). Given that the elevation changes are highly localized to the upper accumulation zones and lower receiving areas of individual glaciers, their contribution to the regional average mass balance rate is negligible compared to the climate‑driven ablation signal observed in this study. In future, combining ice flow velocity data (e.g., ITS_LIVE) and multi‑temporal optical imagery, is needed to more comprehensively assess the impact of surge‑type glaciers on mass balance and the Karakoram anomaly.

**Fig 12:**
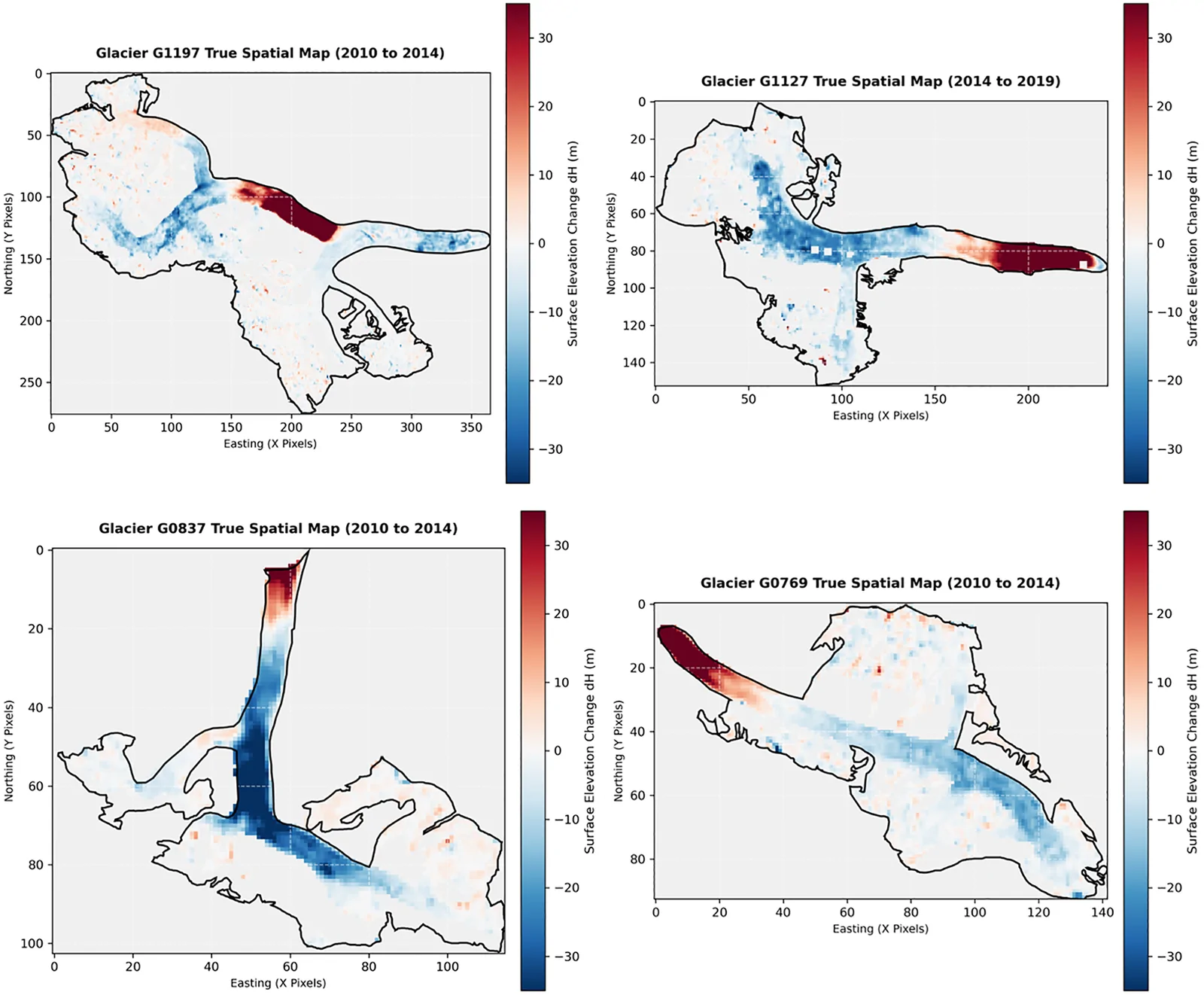
Elevation changes of some surging glaciers in the Karakoram based on ASTER-derived DEM difference analysis.

## 6. Conclusion

This study established a unified reference framework for the fusion of multi-source DEM and altimetry data. By quantifying the phased and high-resolution evolution results of the material balance in the Karakoram region, we revealed the spatio-temporal heterogeneity of the material balance in the Karakoram region and its influencing factors. Our analysis yields the following principal conclusions:

(1) The Karakoram glacier system experienced a slight but statistically significant overall mass loss of −0.0386 ± 0.0032 m w.e. a ^−^ ¹ over the past 25 years. However, the Western Karakoram (WK) and Central Karakoram (CK) sub-regions maintained a near-balanced mass budget, whereas the Eastern Karakoram (EK) showed obvious mass loss, indicating substantial spatial differences in glacier mass balance.
(2) The Karakoram Anomaly evolved from widespread near-equilibrium conditions before 2010 to increasingly intermittent and spatially restricted positive mass balance during 2010–2020. After 2021, the weakening of accumulation-season gains and strengthening of ablation-season losses resulted in increasingly negative net elevation changes, with the extreme melting event in the third quarter of 2022 accounting for approximately 26% of the total mass loss during 2019–2024. Overall, the anomaly weakened substantially at the regional scale after 2020 and persisted only in a limited number of localized basins.
(3) The observed spatio-temporal patterns are driven by a combination of intensifying climatic forcing and topographic modulation. Over the past 25 years, the Karakoram experienced pronounced summer warming accompanied by a significant increase in summer precipitation. These climatic changes were closely associated with the observed glacier mass-balance variability, with enhanced summer warming likely increasing ablation while changes in summer precipitation may have partly modified the accumulation–ablation balance. Topographic characteristics further contributed to the spatial heterogeneity of glacier mass balance, highlighting the combined influence of climatic and topographic factors on glacier responses across the Karakoram.

These findings advance our understanding of the Karakoram anomaly and provided an important reference for the study of other similar regions. With climate warming intensifying, glaciers are encountering increasingly severe environmental conditions, Future work should prioritize the integration of ice-dynamic modeling, direct ice-thickness observations, and high-resolution climate datasets to further elucidate the processes governing glacier retreat and its downstream impacts.

## References

[1] Sorg A, Bolch T, Stoffel M, Solomina O, Beniston M. Climate change impacts on glaciers and runoff in Tien Shan (Central Asia). Nature Clim Change. 2012;2(10):725–31. doi: 10.1038/nclimate1592

[2] Hugonnet R, McNabb R, Berthier E, Menounos B, Nuth C, Girod L, et al. Accelerated global glacier mass loss in the early twenty-first century. Nature. 2021;592(7856):726–31. doi: 10.1038/s41586-021-03436-z 33911269

[3] Kraaijenbrink PDA, Bierkens MFP, Lutz AF, Immerzeel WW. Impact of a global temperature rise of 1.5 degrees Celsius on Asia’s glaciers. Nature. 2017;549(7671):257–60. doi: 10.1038/nature23878 28905897

[4] Yao T, Thompson L, Yang W, Yu W, Gao Y, Guo X, et al. Different glacier status with atmospheric circulations in Tibetan Plateau and surroundings. Nature Clim Change. 2012;2(9):663–7. doi: 10.1038/nclimate1580

[5] Hewitt K. The karakoram anomaly? Glacier expansion and the ‘elevation effect,’ karakoram himalaya. Mt Res Dev. 2005;25: 332–40. doi: 10.1659/0276-4741(2005)025[0332:TKAGEA]2.0.CO;2

[6] Rounce DR, Hock R, Maussion F, Hugonnet R, Kochtitzky W, Huss M, et al. Global glacier change in the 21st century: Every increase in temperature matters. Science. 2023;379(6627):78–83. doi: 10.1126/science.abo1324 36603094

[7] Farinotti D, Immerzeel WW, de Kok R, Quincey DJ, Dehecq A. Manifestations and mechanisms of the Karakoram glacier Anomaly. Nat Geosci. 2020;13(1):8–16. doi: 10.1038/s41561-019-0513-5 31915463 PMC6949123

[8] Matsuo K, Heki K. Time-variable ice loss in Asian high mountains from satellite gravimetry. Earth and Planetary Science Letters. 2010;290(1–2):30–6. doi: 10.1016/j.epsl.2009.11.053

[9] Wang Q, Sun W. Seasonal Cycles of High Mountain Asia Glacier Surface Elevation Detected by ICESat‐2. JGR Atmospheres. 2022;127(23). doi: 10.1029/2022jd037501

[10] Ren S, Zheng D, Zhou Y, Feng M, Guo Y, Li X. The shrinking crown of the tropics: A consistent accelerated trend of glacier retreat since the 1980s. Innov Geosci. 2026;4: 100217. doi: 10.59717/j.xinn-geo.2026.100217

[11] Wang Y, Zheng D, Zhou Y, Nian Y, Ren S, Ren W, et al. Glacier mass change and evolution of Petrov Lake in the Ak-Shyirak massif, central Tien Shan, from 1973 to 2023 using multisource satellite data. Remote Sensing of Environment. 2024;315:114437. doi: 10.1016/j.rse.2024.114437

[12] Millan R, Mouginot J, Rabatel A, Morlighem M. Ice velocity and thickness of the world’s glaciers. Nat Geosci. 2022;15: 124–9. doi: 10.1038/s41561-021-00885-z

[13] Xie F, Liu S, Zhu Y, Qing X, Tan S, Gao Y, et al. Retrieval of high-resolution melting-season albedo and its implications for the Karakoram Anomaly. Remote Sensing of Environment. 2024;315:114438. doi: 10.1016/j.rse.2024.114438

[14] Lehner B, Grill G. Global river hydrography and network routing: baseline data and new approaches to study the world’s large river systems. Hydrol Process. 2013;27: 2171–86. doi: 10.1002/hyp.9740

[15] Liu J, Zhao J, Li Z, Wang Y, Shen S. Elevation change of the Urumqi Glacier No.1 derived from Sentinel-1A data. J Mt Sci. 2021;18: 2656–71. doi: 10.1007/s11629-020-6562-9

[16] Wang S, Alexander P, Wu Q, Tedesco M, Shu S. Characterization of ice shelf fracture features using ICESat-2 – A case study over the Amery Ice Shelf. Remote Sensing of Environment. 2021;255:112266. doi: 10.1016/j.rse.2020.112266

[17] Abdalati W, Zwally HJ, Bindschadler R, Csatho B, Farrell SL, Fricker HA, et al. The ICESat-2 Laser Altimetry Mission. Proc IEEE. 2010;98(5):735–51. doi: 10.1109/jproc.2009.2034765

[18] Muhammad S, Tian L. Mass balance and a glacier surge of Guliya ice cap in the western Kunlun Shan between 2005 and 2015. Remote Sensing of Environment. 2020;244:111832. doi: 10.1016/j.rse.2020.111832

[19] Treichler D, Kääb A, Salzmann N, Xu C-Y. Recent glacier and lake changes in High Mountain Asia and their relation to precipitation changes. The Cryosphere. 2019;13(11):2977–3005. doi: 10.5194/tc-13-2977-2019

[20] Markus T, Neumann T, Martino A, Abdalati W, Brunt K, Csatho B, et al. The ice, cloud, and land elevation Satellite-2 (ICESat-2): Science requirements, concept, and implementation. Remote Sensing of Environment. 2017;190:260–73. doi: 10.1016/j.rse.2016.12.029

[21] Smith B, Fricker HA, Holschuh N, Gardner AS, Adusumilli S, Brunt KM, et al. Land ice height-retrieval algorithm for NASA’s ICESat-2 photon-counting laser altimeter. Remote Sensing of Environment. 2019;233:111352. doi: 10.1016/j.rse.2019.111352

[22] Toutin T. ASTER DEMs for geomatic and geoscientific applications: a review. International Journal of Remote Sensing. 2008;29(7):1855–75. doi: 10.1080/01431160701408477

[23] Crippen R, Buckley S, Agram P, Belz E, Gurrola E, Hensley S, et al. Nasadem global elevation model: Methods and progress. Int Arch Photogramm Remote Sens Spatial Inf Sci. 2016;XLI-B4:125–8. doi: 10.5194/isprs-archives-xli-b4-125-2016

[24] Raup B, Racoviteanu A, Khalsa SJS, Helm C, Armstrong R, Arnaud Y. The GLIMS geospatial glacier database: A new tool for studying glacier change. Global and Planetary Change. 2007;56(1–2):101–10. doi: 10.1016/j.gloplacha.2006.07.018

[25] Pfeffer WT, Arendt AA, Bliss A, Bolch T, Cogley JG, Gardner AS, et al. The randolph glacier inventory: A globally complete inventory of glaciers. J Glaciol. 2014;60: 537–52. doi: 10.3189/2014JoG13J176

[26] Muñoz-Sabater J, Dutra E, Agustí-Panareda A, Albergel C, Arduini G, Balsamo G, et al. ERA5-Land: a state-of-the-art global reanalysis dataset for land applications. Earth Syst Sci Data. 2021;13(9):4349–83. doi: 10.5194/essd-13-4349-2021

[27] Liu L, Gu H, Xie J, Xu Y. How well do the ERA‐Interim, ERA ‐5, GLDAS ‐2.1 and NCEP‐R2 reanalysis datasets represent daily air temperature over the Tibetan Plateau? Int J Climatol. 2021;41: 1484–505. doi: 10.1002/joc.6867

[28] Li C, Jiang L, Liu L, Wang H. Regional and altitude-dependent estimate of the SRTM C/X-band radar penetration difference on high mountain asia glaciers. IEEE J Sel Top Appl Earth Observations Remote Sensing. 2021;14:4244–53. doi: 10.1109/jstars.2021.3070362

[29] Girod L, Nuth C, Kääb A, McNabb R, Galland O. MMASTER: Improved ASTER DEMs for Elevation Change Monitoring. Remote Sensing. 2017;9(7):704. doi: 10.3390/rs9070704

[30] Gu L, Che Y, Zhang M, Chen L, Wu J. A seasonal estimate of the Kumkol Basin glacier mass balance by satellite laser altimeter data and ASTER GDEM. J Hydrol Reg Stud. 2025;61: 102648. doi: 10.1016/j.ejrh.2025.102648

[31] Nuth C, Kääb A. Co-registration and bias corrections of satellite elevation data sets for quantifying glacier thickness change. The Cryosphere. 2011;5(1):271–90. doi: 10.5194/tc-5-271-2011

[32] Dehecq A, Millan R, Berthier E, Gourmelen N, Trouve E, Vionnet V. Elevation Changes Inferred From TanDEM-X Data Over the Mont-Blanc Area: Impact of the X-Band Interferometric Bias. IEEE J Sel Top Appl Earth Observations Remote Sensing. 2016;9(8):3870–82. doi: 10.1109/jstars.2016.2581482

[33] Li C, Jiang L, Liu L, Wang H. Regional and altitude-dependent estimate of the srtm c/x-band radar penetration difference on high mountain asia glaciers. IEEE J Sel Top Appl Earth Observations Remote Sensing. 2021;14:4244–53. doi: 10.1109/jstars.2021.3070362

[34] Huss M. Density assumptions for converting geodetic glacier volume change to mass change. The Cryosphere. 2013;7(3):877–87. doi: 10.5194/tc-7-877-2013

[35] Nuth C, Kohler J, Aas HF, Brandt O, Hagen JO. Glacier geometry and elevation changes on svalbard (1936–90): A baseline dataset. Ann Glaciol. 2007;46: 106–16. doi: 10.3189/172756407782871440

[36] Neelmeijer J, Motagh M, Bookhagen B. High-resolution digital elevation models from single-pass TanDEM-X interferometry over mountainous regions: A case study of Inylchek Glacier, Central Asia. ISPRS J Photogram Remote Sens. 2017;130:108–21. doi: 10.1016/j.isprsjprs.2017.05.011

[37] Gardelle J, Berthier E, Arnaud Y, Kääb A. Region-wide glacier mass balances over the Pamir-Karakoram-Himalaya during 1999–2011. The Cryosphere. 2013;7(4):1263–86. doi: 10.5194/tc-7-1263-2013

[38] Brun F, Berthier E, Wagnon P, Kääb A, Treichler D. A spatially resolved estimate of High Mountain Asia glacier mass balances, 2000-2016. Nat Geosci. 2017;10(9):668–73. doi: 10.1038/NGEO2999 28890734 PMC5584675

[39] Fan Y, Ke C-Q, Zhou X, Shen X, Yu X, Lhakpa D. Glacier mass-balance estimates over High Mountain Asia from 2000 to 2021 based on ICESat-2 and NASADEM. J Glaciol. 2023;69: 500–12. doi: 10.1017/jog.2022.78

[40] Shean DE, Bhushan S, Montesano P, Rounce DR, Arendt A, Osmanoglu B. A systematic, regional assessment of high mountain asia glacier mass balance. Front Earth Sci. 2020;7. doi: 10.3389/feart.2019.00363

[41] Lhakpa D, Fan Y, Cai Y. Continuous Karakoram Glacier Anomaly and its response to climate change during 2000–2021. Remote Sens. 2022;14: 6281. doi: 10.3390/rs14246281

[42] Zhou S, Sun Z, Abowarda AS, Aletoum E, Huang Q, Lin X, et al. Has the Karakoram anomaly persisted over the past two decades? GIScience Remote Sens. 2025;62: 2548059. doi: 10.1080/15481603.2025.2548059

[43] Wang G-Y, Ke C-Q, Fan Y-B, Shen X-Y, Cai Y, Nourani V. Accelerated glacier mass loss in the mid-latitude Eurasia from 2019 to 2022 revealed by ICESat-2. Advances in Climate Change Research. 2024;15(5):815–29. doi: 10.1016/j.accre.2024.09.008

[44] Zhou S, Sun Z, Xie J, Zhao F, Huang K, Wang H, et al. Investigating glacier mass balance and driving factors of the karakoram anomaly. J Hydrol Reg Stud. 2025;61: 102616. doi: 10.1016/j.ejrh.2025.102616

[45] Bhattacharya A, Bolch T, Mukherjee K, King O, Menounos B, Kapitsa V, et al. High Mountain Asian glacier response to climate revealed by multi-temporal satellite observations since the 1960s. Nat Commun. 2021;12(1):4133. doi: 10.1038/s41467-021-24180-y 34226559 PMC8257854

[46] Wu K, Liu S, Jiang Z, Xu J, Wei J, Guo W. Recent glacier mass balance and area changes in the Kangri Karpo Mountains from DEMs and glacier inventories. The Cryosphere. 2018;12(1):103–21. doi: 10.5194/tc-12-103-2018

[47] Zhao F, Long D, Li X, Huang Q, Han P. Rapid glacier mass loss in the Southeastern Tibetan Plateau since the year 2000 from satellite observations. Remote Sensing of Environment. 2022;270:112853. doi: 10.1016/j.rse.2021.112853

[48] Zhu F, Yao T-D, Zhu M-L, An L, Wang P, Huang G-F, et al. The role of mid-latitude zonal wind anomalies in extreme glacier mass loss on central Tibetan Plateau. Adv Climate Change Res. 2026;17(4):768–79. doi: 10.1016/j.accre.2026.03.010

[49] Khadka A, Wagnon P, Brun F, Shrestha D, Lejeune Y, Arnaud Y. Evaluation of ERA5-Land and HARv2 Reanalysis Data at High Elevation in the Upper Dudh Koshi Basin (Everest Region, Nepal). J Appl Meteorol Climatol. 2022;61: 931–54. doi: 10.1175/JAMC-D-21-0091.1

[50] Winiger M, Gumpert M, Yamout H. Karakorum–Hindukush–western Himalaya: assessing high‐altitude water resources. Hydrol Process. 2005;19: 2329–38. doi: 10.1002/hyp.5887

[51] Yao T, Thompson L, Yang W, Yu W, Gao Y, Guo X, et al. Different glacier status with atmospheric circulations in Tibetan Plateau and surroundings. Nature Clim Change. 2012;2(9):663–7. doi: 10.1038/nclimate1580

[52] Lovell H, Benn DI, Jiskoot H, Stokes CR, Flowers GE, Guillet G, et al. Glacier surging and surge-related hazards in a changing climate. Nat Rev Earth Environ. 2026;7(3):162–80. doi: 10.1038/s43017-025-00757-9

